# Elements of Suffering in Myalgic Encephalomyelitis/Chronic Fatigue Syndrome: The Experience of Loss, Grief, Stigma, and Trauma in the Severely and Very Severely Affected

**DOI:** 10.3390/healthcare9050553

**Published:** 2021-05-09

**Authors:** Patricia A. Fennell, Nancy Dorr, Shane S. George

**Affiliations:** 1 Albany Health Management Associates, Inc., Albany, NY 12203, USA; 2 Department of Psychology, The College of Saint Rose, Albany, NY 12203, USA; dorrn@strose.edu (N.D.); georges947@strose.edu (S.S.G.)

**Keywords:** ME/CFS, Chronic Fatigue Syndrome, severely and very severely ill, trauma, grief, chronic illness, fatigue, suicide, stress, uncertainty

## Abstract

People who are severely and very severely affected by Myalgic Encephalomyelitis/Chronic Fatigue Syndrome (ME/CFS) experience profound suffering. This suffering comes from the myriad of losses these patients experience, the grief that comes from these losses, the ongoing stigma that is often experienced as a person with a poorly understood, controversial chronic illness, and the trauma that can result from how other people and the health care community respond to this illness. This review article examines the suffering of patients with ME/CFS through the lens of the Fennell Four-Phase Model of chronic illness. Using a systems approach, this phase framework illustrates the effects of suffering on the patient and can be utilized to help the clinician, patient, family, and caregivers understand and respond to the patient’s experiences. We highlight the constructs of severity, uncertainty, ambiguity, and chronicity and their role in the suffering endured by patients with ME/CFS. A composite case example is used to illustrate the lives of severely and very severely affected patients. Recommendations for health care providers treating patients with ME/CFS are given and underscore the importance of providers understanding the intense suffering that the severely and very severely affected patients experience.

## 1. ME/CFS: The Severely and Very Severely Affected

Understanding the depth and breadth of the suffering experienced by people who are severely and very severely affected by Myalgic Encephalomyelitis/Chronic Fatigue Syndrome (ME/CFS) is necessary in order to adequately care for them. These patients exhibit heterogeneous symptoms in multiple body systems and life domains. Their symptoms typically include fatigue, post-exertional malaise (PEM), sleep disturbance, cognitive impairment, orthostatic intolerance, muscle and/or joint pain, as well as flu-like symptoms including headaches, sore throat, swollen lymph glands, chills, and night sweats [1]. Commonly, patients also have bowel disorders, allergies, chemical sensitivities, light and sound disturbance, shortness of breath, and irregular heartbeat, etc. [1]. These symptoms fluctuate in kind, duration, frequency, and intensity over the lifetime of those afflicted [2,3,4,5,6,7]. In common with other chronic illnesses, these symptom impairments can affect multiple domains in patients’ lives—the physical, the psychological, and the social-interactive [2,3,8,9]. Given the profound ongoing uncertainty, ambiguity and chronicity of ME/CFS, patients subsequently experience significant suffering. This suffering stems not only from the devastating physical symptoms, but also from ongoing stigma, loss, grief, and trauma. Through the lens of a phase model of chronic illness, the current paper examines these varieties of suffering. This phase framework illustrates the effects of suffering on the patient and can be utilized to help the clinician, patient, family, and caregivers understand and respond to the patient’s experiences. A composite case example is used to illustrate the lives of severely and very severely affected patients.

The International Consensus Criteria categorize patient functionality, according to reductions from their premorbid levels, as satisfying the criteria for either a severe subgroup when they are rendered mostly bedbound or a very severe subgroup when they are rendered totally bedbound and require assistance with basic functions and tasks of daily living [10]. Symptom severity in patients with ME/CFS can be difficult to ascertain. Commonly, severity is assessed through the patient’s ability to function with their condition. While functionality can vary between patients depending on the symptoms that are most predominant, research supports the persistence of physical and psychological fatigue severity, and primary symptoms, such as post-exertional malaise, cognitive dysfunctions, gastrointestinal issues, and impaired sleep, in maintaining reduced functionality [6,11]. Given that patients who have experienced severe reductions in functioning that render them homebound or bedbound account for approximately 25–29% of ME/CFS patients [12], there is a need for increased attention toward this subgroup.

## 2. Fennell Four-Phase Model: A General Description

Several models have been developed to describe the process of living with chronic illness that are applicable to patients with ME/CFS [2,13,14,15,16]. Fennell’s Four-Phase Model [2] is useful for defining and describing four phases that occur in ME/CFS. This model has been thoroughly developed over the last several decades [2,3,17,18,19,20,21,22] through clinical encounters, patient testimonies, and empirical research [5,7,23,24,25]. The model explicitly captures the changing experience of patients over time in all domains of their lives—their physical, psychological, and social-interactive worlds.

Phase 1 of Fennell’s Four-Phase Model is a state of chaos and crisis. It lasts from the onset of the disease through an emergency state when patients typically seek medical help. The phase usually concludes when patients receive a diagnosis or when their symptoms become stable and recognizable to them. Phase 2 is a time of stabilization. Initially, patients attain a plateau of symptoms. They become familiar with the symptoms and achieve some success in coping with them. Patients begin to feel a degree of control returning to their lives. In Phase 3, characterized by resolution, patients acknowledge that they have a chronic illness, and that their lives have thus been changed forever. With this recognition, however, can come existential despair. If patients are able to develop meaning in their lives, they begin to construct a new self that deals with the practical aspects of their illness; yet, they are not overwhelmed or totally preoccupied with it. Phase 4 is one of integration. Patients solidify their newly constructed self and reach out into the world again to participate as fully as they can in as complete a life as their physical condition permits.

Patients ordinarily proceed through the phases in sequence, but as in non-hierarchical stage models, they usually slip back into a prior phase or recycle, sometimes several times [26,27]. Attainment of integration (Phase 4) is not a permanent condition. Crises, especially those brought about by non-ME/CFS illnesses, accidents, or personal tragedies, can throw patients back into the chaos of Phase 1.

Patients present differing symptomatology during each phase and thus respond differently during specific instances of clinical interviews and data collection over time. If these responses are collapsed across phases, important distinctions about experience of the illness can be lost, which in turn may distort or obscure understanding of the illness [5].

The following section describes the course of ME/CFS using the Four-Phase Model to help illustrate how patients present in the physical-behavioral, the psychological, and the social-interactive domains during each phase of the model. The general description of the three domains in each phase includes a table outlining its characteristics. A composite case history illustrates how these generalizations manifest in actual patients, focusing on those who are severely and very severely affected.

## 3. ME/CFS through the Lens of the Four-Phase Model

### 3.1. Phase 1: Crisis

#### 3.1.1. Physical-Behavioral Domain

The physical functioning of a person with ME/CFS in Phase 1 occurs in a linear progression through three periods (see Table 1). The patient copes or contends with the symptoms in the first period. Patients experience varying levels of severity and combinations of the aforementioned symptoms, including fatigue, flu-like symptoms, increasing cognitive confusion, muscle and/or joint pain, and non-restorative sleep. ME/CFS can have a gradual or a sudden onset. Since the onset of ME/CFS can frequently occur following an illness such as flu or a respiratory infection, patients may attribute their ME/CFS symptoms to a very slow recovery. They may sometimes try to ignore them.

Next, patients enter a more intense onset period, where their symptoms more insistently demand their attention. It becomes more and more difficult for the patients to believe that (a) the symptoms are unrelated to the prior illness, or (b) they may be suffering a new or unresolved infection, or (c) they may be experiencing the symptoms of a different illness entirely. After a varying period of time, depending on the severity of symptoms, patients enter an acute emergency period and almost always seek medical help, even if they have not done so previously.

With most chronic illnesses, patients receive a reasonably firm diagnosis at the end of Phase 1. ME/CFS can be more problematic. It is poorly understood, and less familiar to many primary care physicians than illnesses such as rheumatoid arthritis or multiple sclerosis. The diagnostic process requires the presence of several symptoms occurring simultaneously as well as the exclusion of other illnesses that share some of the same symptoms. One of the defining symptoms—profound fatigue—needs to be ongoing for at least 6 months for patients to satisfy some ME/CFS case definitions. This particular diagnostic parameter in itself can mean that patients may live for a considerable time in crisis with debilitating—sometimes very debilitating—symptoms before they receive a diagnosis.

#### 3.1.2. Psychological Domain

Severity of presentation of symptoms may impact the length of time between the first period of coping and clinically observed onset. Patients who have a lengthy onset—and ME/CFS patients often wait a long time for diagnosis [13]—may use denial as a coping mechanism [28]. Since they often receive little recognition and support from either the health care community or society at large, ME/CFS patients, especially those with a lengthy onset, may act with others in their life to deny their symptoms in order to hopefully maintain their daily lives. Stormorken and colleagues [13] suggest improvement can be delayed when patients engage in denial and do not accept that they are ill.

Typically in Phase 1, ME/CFS patients regularly report receiving conflicting advice and sometimes disbelief from health care professionals [29]. The patients do not know how to describe their condition to themselves or others [15]. They may feel emotional isolation, fear of others, mood swings, and intense confusion. They may also feel shame from the act of reporting such difficult symptoms and being disbelieved. They can also feel embarrassed if symptoms such as fatigue, cognitive impairment, and lack of energy are displayed in front of other people [13].

If their condition continues to deteriorate, denial can give way to intrusive feelings of fear, self-hatred, despair, and disorientation. As is the case with other diseases [30,31], some patients with ME/CFS report that they feel they are somehow responsible for what is happening to them, but they do not believe they can do anything about it.

By the time they contact their doctor, many ME/CFS patients present with urgency. They usually hold the locus of treatment and cure to be totally outside themselves. Similar to those with other chronic illnesses [32,33], patients with ME/CFS suffer from fear and shame, experience intrusive ideations about dying, but can sometimes exhibit a high degree of denial about their psychological suffering. 

Homebound and bedbound ME/CFS patients experience an abundance of losses of their former selves due to their reduced functionality. Wiborg and colleagues [34] found that patients who were homebound had more impairment in home management, mobility, and functionality, as well as reporting an increased amount of physical problems as compared to other patients with ME/CFS. Homebound patients further struggled to hold paying jobs and thus experienced financial losses as well. The cumulative effect of all these physical losses frequently produces intense psychological suffering in patients with severe deficits in functionality [34,35,36].

Patients with severe or very severe ME/CFS disabilities experience a greater exacerbation of symptoms as losses of their physical capacities accumulate, and they can subsequently develop psychological symptoms as a result. Exacerbation of symptoms may compound when their psychological symptoms produce losses in their social life. These psychological symptoms, often appearing depression- or anxiety-like, may not be separate clinical diagnoses, but distinct emotional responses to the physical losses associated with a debilitating chronic illness [37]. Dancey and Friend [38] posit the importance of recognizing the intrusiveness of ME/CFS when assessing its psychological impact on patients. Their results suggest ME/CFS is an extremely intrusive illness that disrupts more aspects of patients’ lives to a greater extent than does multiple sclerosis, laryngeal cancer, end-stage renal disease, irritable bowel syndrome, rheumatoid arthritis, and insomnia. As such, while depressive and anxious symptoms can result from the severity of the patient’s condition, they are at least partially mediated by the intrusiveness of that condition. ME/CFS patients who are severely and very severely affected can thus experience greater psychological suffering as their reduced mobility, fatigue, impaired sleep, and cognitive dysfunctions intrude on more aspects of their life.

ME/CFS patients can also experience trauma. Research supports the existence of trauma symptoms in individuals who experience illness processes [39,40], and these can result from a variety of factors. Patients can experience stigmatization from a variety of sources, such as from their community, health care professionals, the media, and the public, and this can be traumatic. Their illnesses, often lacking in observable or measurable symptomatology, may be regarded as character failings rather than legitimate threats to well-being [41,42,43].

When these difficult to observe symptoms initially appear, patients who often cannot identify what they are experiencing have little or no tolerance for the uncertainty and ambiguity of their condition. Uncertainty is a primary element in illness experiences [44,45,46,47] and has been described as having a negative impact on how patients cope and their disease outcomes. The evidence suggests uncertainty creates suffering in a variety of chronic illnesses [48,49,50,51,52]. ME/CFS patients can experience uncertainty on three levels. Firstly, they can experience the uncertainty of the present and the unknowable future regarding all the symptoms they endure. Secondly, patients can experience uncertainty from their providers of care who may also be struggling with the unknowns and the unknowable regarding their patients’ health outcomes and future [53]. Lastly, they suffer uncertainty from their culture and communities regarding the legitimacy, acceptability, and even the reality of their condition [53].

Not only does uncertainty play a role in the crisis phase, but ambiguity does as well [22]. The provider and the patient may be ruling out competing diagnoses and be beginning to suspect a tentative diagnosis of ME/CFS might be appropriate. However, what to do about an ME/CFS diagnosis is also ambiguous: determining the best course of action for this is always problematic because of the range of possible causes, assessments, and feasible treatments. 

#### 3.1.3. Social-Interactive Domain

When ME/CFS patients can no longer hide their symptoms from others, they find that some of their friends, family members, acquaintances, coworkers, and health care providers regard them as malingering or mentally ill. These others may think the ME/CFS patients are trying to avoid work [54], which increases the burden on everyone around them. The disbelief and suspicion that ME/CFS patients sometimes encounter can make them very cautious. These patients may be afraid to express how they actually feel or may misrepresent how they feel. Simultaneously, they may withdraw emotionally from others to avoid further rejection and negative stereotyping.

Some ME/CFS patients continue to try to work during Phase 1, which often creates difficulties with coworkers and supervisors. Increasingly, they may be late to work and unable to complete tasks in a timely manner. Eventually they are usually absent a great deal. Initial concern on the part of fellow workers can, therefore, give way to irritation and resentment as others have to take up the patients’ workload. Some patients use their sick leave so quickly that they have to take unpaid leave or attempt to get disability. Disability may be difficult to obtain at this phase of the illness.

Three critical issues become evident during Phase 1. First, ME/CFS patients can be traumatized by the physical, psychological, and social impact of the acute emergency period [55,56,57,58,59,60]. Second, their friends, family, coworkers, and clinicians can be vicariously traumatized by what is happening [8,61,62,63,64,65,66,67,68,69]. Third, these significant others begin to queue up on a continuum that extends from suspicion to support in response to the ME/CFS patient’s observable, decreased participation in activities. These social responses are often negative, if not in fact, stigmatizing, and can cause ME/CFS patients further secondary traumatization [2,3,70]. It is possible to propagate or mitigate ongoing traumatization. For example, the level of supervision and support in the health care organizations employing the clinicians can affect traumatization [71]. An effective health care organization provides support and supervision to providers who experience the daily grind of chronic suffering so they can avoid vicarious traumatization, subsequent burnout, and inflicting unintentional iatrogenic traumatization. The maturation, premorbidity, and comorbidity of the patients’ social network also affect traumatization [55,72,73,74]. A supportive social network can tolerate the disruption of work and social exchange that the emergency period brings while containing and buffering it for the patients.

### 3.2. Elizabeth’s Story: Phase 1

#### 3.2.1. Physical-Behavioral Domain

Elizabeth is a married white woman in her late 30s. She has two children, Eva, age 13, and Michael, age 11. Jim, her husband, is a sales consultant, which often requires him to work out of town for one or two weeks at a time. Elizabeth works at a medical practice in the suburb where the family lives.

Over the past several months, Elizabeth has been increasingly distracted by a number of physical symptoms that are beginning to frighten her because they are interfering with her life and her work. She is exhausted most of the time and is not sleeping well. She thinks, at first, that she is just not completely recovered from a recent respiratory infection, but months are passing, and she still feels completely drained. Elizabeth has never been sick with more than occasional colds, so she rarely sees the doctor. At first, she does not think her situation is very different from that of a lot of her friends, because all the working mothers she knows are always tired, too. She just keeps trying to carry on with her regular activities, snatching whatever moments she can to nap or at least rest. However, as time is passing, she is finding it more and more difficult to get dressed, drive or work for any length of time when she arrives at her job.

Elizabeth is just entering Phase 1. Physically and behaviorally, Elizabeth is attempting to cope with her symptoms. Even though she feels very unwell, she tries to ignore or “push through” her symptoms, to push them out of her consciousness, and to continue her regular activities.

Eventually, however, Elizabeth’s exhaustion, increased muscle pain, and headaches make it impossible for her to ignore her symptoms; she has trouble climbing the few steps into her front door. Now she is entering the acute onset period. Elizabeth decides to go see her primary care physician.

The doctor listens to Elizabeth describe her symptoms and gives her a physical examination. They talk a bit about her new limitations at work and at home. The doctor tells Elizabeth that test results do not reveal anything physically wrong with her. He suggests her exhaustion may be due to her poor sleep. He wonders if the many demands of her job and family life may be causing her difficulties. He also thinks that she may be mildly depressed, but not enough to require medication. He wants to follow up with her in six weeks and recommends that Elizabeth relax, try to get to bed earlier, cut back at work, and perhaps join an exercise class to help relieve her symptoms.

Elizabeth wants to follow the doctor’s suggestions, but she does not dare cut back at work any more than she already has because the family needs the income from her job. Additionally, she cannot imagine how she could take an exercise class given her increasing difficulty ambulating from the bedroom to the bathroom. Additionally, it is not so much that she gets to bed late as that she wakes frequently at night and cannot get back to sleep again because her body aches and she feels as if she is oddly moving or vibrating. Her symptoms progressively worsen. Not only is she extremely fatigued, she experiences more and more trouble thinking. She is having memory problems, word finding, and concentration difficulty. She became tearful when she could not remember her home address when asked.

Elizabeth is now entering the acute emergency period. After six weeks, Elizabeth returns to her primary care physician who orders additional blood tests and refers her to a psychiatrist to assess her stress level and rule out clinical depression. The psychiatrist reports that Elizabeth appears to be suffering from reactive depression in response to her physical condition. The blood work results raise questions that cause the primary care doctor to refer Elizabeth to a rheumatologist. For months, she is examined, tested, and enters the diagnostic limbo of uncertainty and ambiguity. It is not until almost a year later that the rheumatologist is able to give Elizabeth a tentative diagnosis of Myalgic Encephalomyelitis or Chronic Fatigue Syndrome. Even now, some of the doctors are not really sure it is ME/CFS.

However, having a diagnosis, even a tentative one, makes an enormous difference to Elizabeth, for it finally gives her a way to at least partially understand and describe her experiences to herself and others.

#### 3.2.2. Psychological Domain

During Elizabeth’s lengthy coping and subsequent onset periods, she uses denial as a coping mechanism. Denial comes into play after her initial visit to her primary care physician, who tells her that he suspects she is mildly depressed and suffering from stress. She wants to believe that is an accurate diagnosis and so she agrees with her doctor, her husband, and the people at work that it is possible for her to return to her daily life.

However, as Elizabeth’s symptoms worsen, other feelings begin to intrude. Like many people, Elizabeth has constructed two selves—a private persona and a public persona. Additionally, like others, Elizabeth reveals more or less of her private persona to individuals in her life depending on how intimate she is with them and what particular situation she is in. As Elizabeth’s condition continues to deteriorate, she finds that her private persona is beginning to intrude on her public persona in ways that she cannot control. One day at a virtual staff meeting with the medical practice (where she now works part-time in billing from home, as meetings with her have to be virtual with her out of the office), Elizabeth suddenly bursts into tears. She feels embarrassed that she may have made her superior and coworkers feel uncomfortable, and as they comfort her, she feels like a burden to them. This behavior is not the self that Elizabeth recognizes. She cannot identify why she feels the way she does, and because of her uncertainty regarding her health, she feels as if she is losing control. She feels shame about her loss of control, and increased fear and despair about the uncertainty and ambiguity of her condition. She knows she feels terrible physically and is getting worse, and she worries she could actually be dying; at the same time, she feels dissociated, and she wonders if she could be losing her mind. It is important to remember that at this point no one has yet given Elizabeth’s situation a definitive label.

Elizabeth has no effective way to express how she is feeling and when she tries, the people she talks to can only make up explanations and suggestions for improvement based on their own personal experiences, not on an understanding of what is happening to her. Elizabeth feels increasingly isolated because she fears what is happening to her and what other people will think of her. She is particularly afraid to talk to the person who used to be closest to her—her husband, Jim. Elizabeth and Jim have already been having marital difficulties due to conflicts over money and the amount of time Jim needs to be away from home. Elizabeth feels that Jim, by default, gets out of his fair share of home and child care duties. She struggles with basic activities of daily living and she increasingly relies upon her children’s help. She fears she cannot quit her job, because the family cannot make ends meet without her part-time salary. In any case, she likes her job, which she used to do very well. She has received a lot of praise at work, especially from her supervisor, and she and Jim had both been hoping that she would get a promotion. The new position would have brought in more money, but Elizabeth would have to work full time and now that would be impossible.

In part, as a consequence of her pain, her fears, the lack of useful information about her condition, and her growing isolation, Elizabeth now begins to suffer emotionally with grief and anger from her suffering and losses. Half the time she is in tears, she says, and the other half she is furious. Jim has tried to be sympathetic, but now he is getting frustrated. The children act scared of her and disappear whenever possible. Even Elizabeth’s coworkers find her muddled and distracted, whereas she used to be focused and attentive. Elizabeth is losing her life as she has known it, and she is frightened she will never get it back.

#### 3.2.3. Social-Interactive Domain

Many of Elizabeth’s psychological mechanisms and reactions result directly from what is happening in her social-interactive life. During the coping and onset periods, Elizabeth’s family, friends, and coworkers respond in various ways to her experiences. They notice only that she is tired a lot of the time and missing work and for a while, they are sympathetic. She is a hard worker, and her female coworkers, particularly, have a strong personal understanding of how hard it is to juggle a job, home, and children. As Elizabeth accomplishes less and misses more and more work, however, they become critical. They have difficult lives too, but they manage to come to work, and they accomplish their assigned tasks.

Elizabeth’s children think that she is acting strange. She does not behave like the mother they are used to. Jim finds her unpredictable and emotionally extreme. He is used to hearing Elizabeth say she is tired, but her complaints seem so serious that Jim is genuinely worried and urges her to go to the doctor. The doctor’s diagnosis of suspected depression and stress seems reasonable to Jim, and he makes an effort to help more by staying in town or taking only short jobs away from home. However, that cannot go on forever, and Elizabeth does not seem to change. He thinks she could do a lot more if she tried, but she seems just to complain or sleep.

During the acute emergency period, while Elizabeth is being examined and tested extensively, Jim sometimes wonders whether anything is actually wrong with Elizabeth—maybe it is “all in her head”. Some of her coworkers feel that way, too. Maybe Elizabeth is having some kind of emotional breakdown.

Finally, Elizabeth gets her diagnosis, however tentative, of ME/CFS. Although this gives her the relief of a name and an explanation, she now finds that the illness has put her squarely on the forefront of a cultural debate. Caught in a mesh of divergent popular beliefs regarding her partially understood disease, Elizabeth finds that some of her friends and coworkers—even some of the medical personnel she sees—view her negatively. For the first time in her life, Elizabeth begins to experience rejection by the society at large. She becomes very cautious about expressing her fears or revealing her pain because she does not want others to withdraw from her. Not only is she afraid of other people now, but her physical condition itself interferes with her reaching out socially.

Elizabeth’s home life was already stressed prior to the onset of her illness, and after enduring the coping, onset, and acute emergency periods, her illness has greatly exacerbated the situation. At the medical practice, Elizabeth’s immediate supervisor is sympathetic because the supervisor’s sister has fibromyalgia, can no longer work, and is on disability. While lacking an understanding of the complexities of Elizabeth’s condition, her supervisor is sympathetic to Elizabeth’s problems and wants to help. Upper levels of management, however, think that Elizabeth should probably be replaced, and it is unclear how effective the supervisor’s advocacy will be. Fortunately, on the medical front, Elizabeth’s primary physician has become very involved with her case but cannot spend as much time as he would like to talk with Elizabeth and must focus on assessment and treatment of her acute physical symptoms. Time does not allow the doctor to discover in depth how Elizabeth is feeling or how she is coping with the whole illness experience. A few family members and friends are wondering whether she should consider filing for disability.

### 3.3. Phase 2: Stabilization

#### 3.3.1. Physical-Behavioral Domain

As ME/CFS patients enter Phase 2, they usually proceed physiologically to a plateau. Their specific symptoms and their severity determine the functional level of their plateau. Their fatigue intensity follows predictable patterns, their muscle-joint pain remains stable or increases, they begin to recognize when they will have greater or lesser cognitive function, and so forth. During this period, symptoms stabilize and assume a somewhat familiar pattern. This in turn can help orient patients cognitively and psychologically. Table 2 lists the periods and characteristics of Phase 2.

#### 3.3.2. Psychological Domain

When ME/CFS patients finally receive a diagnosis, they often feel an initial sense of profound relief [16,75]. Getting a name for their experience demystifies some of the disturbing uncertainties about their symptoms. Patients strongly desire to understand their illness experience [76,77], and even when ME/CFS patients do not get a diagnosis, they can begin to feel some increasing control when they discern a pattern in their symptoms and discover relationships between activities and symptoms. At the same time, their self-pathologizing and intrusive ideations usually decrease.

During both Phase 1 and Phase 2, ME/CFS patients can experience stigmatization, rejection, and iatrogenic traumatization [43,55,60,78,79,80,81,82]. As a result, they and their families may censor what they say and to whom. Patients often pretend to be well; they may attempt to "pass" for normal, but this presents a double-edged sword. While patients that pass may appear "normal," their symptoms persist and they face increased social pressure to conform in spite of their disabling symptoms [59]. When patients do present visible symptoms, they are more apt to experience internalized stigma, and may feel pressured to hide their condition, fearing that their condition may be exposed [41,43]. Thus, they frequently withdraw from hurtful social contacts, and when possible, they seek others with ME/CFS [2,3]. 

The initial relief occasioned by diagnosis usually fades quickly. As ME/CFS patients discover that their condition is not widely understood and that no treatment options promise a cure, they often begin searching for clinicians who can help them. This seeking behavior is natural to Phase 2 and, in many ways, can be a sign of emotional health. The patients are attempting to exert control over their suffering and to reject the disempowerment they experienced in Phase 1. However, confusion, urgency, and desperation can intensify as they consult with medical providers and encounter the ambiguity of conflicting opinions, assuming they are even well enough to attend medical visits. Some ME/CFS patients report a general lack of support, guidance, and knowledge from health care providers and clinicians when they try to find out more about their illness [75]. Indeed, without biomedical tests for diagnosis, patients can feel they are fighting to persuade their physician (and others) they are physically ill [76]. In addition, patients and their families often encounter disbelief of their reported symptoms by their physicians [83]. This results in the patients feeling socially disqualified and devalued. Not being believed is a form of stigma, in which they experience a subsequent loss of identity as well [84,85,86].

Patients may be traumatized by their clinicians when their condition is dismissed or trivialized because they perceive their patient-physician relationship is being “betrayed” [87]. The prevalence of uncertainty and ambiguity can also impact patients’ propensity to continue to seek treatment [88], and can engender distrust toward health care professionals [89].

ME/CFS patients are also struggling against their new physical and cognitive limitations in Phase 2. They do not always know the limits of what they can do on a given day and can be confused about how and where to set boundaries. They can no longer perform as they used to, but familial and community pressures, to say nothing of their own internal desires, make them attempt to maintain their former roles and schedules. As the severely and very severely affected may repeatedly fail to live up to their former roles and expectations, their feelings of guilt and shame heighten, together with an increasing sense of purposelessness, worthlessness, and anomie.

ME/CFS patients gradually learn that they can no longer do “normal” tasks such as shopping or cleaning. The severely affected and very severely affected patients learn they cannot walk upstairs, drive, sit up in bed for very long, or have a normal social conversation without physical consequences. They sometimes cannot perform essential tasks such as paying bills. Similar to a stroke survivor, they are not completely confident about how their body, brain, or emotions will behave in any given situation.

As ME/CFS patients progress through Phase 2, they start to understand the relationship between their activities and their symptoms. With appropriate guidance, they also develop insight into their own attitudes and those of the people around them. Maintaining insight is notoriously difficult. For ME/CFS patients without strong clinical and social support, it can be easy to turn to destructive anodynes such as alcohol or drugs, which will usually spiral the patient back into a Phase 1 crisis. This can also happen when patients work beyond their capacity in an attempt to behave as they did before they became ill. Some patients never really leave Phase 1, while others endlessly cycle between Phase 1 and Phase 2 [2,3]. They are not able to achieve the acknowledgment of chronicity that comes during Phase 3 because they cannot tolerate the implications of permanent illness.

Physical disruption and the possibility of permanent illness can act as the catalyst for the loss of a normal life [84,86,90,91]. Losses rapidly accumulate when these physical disruptions bleed into the patients’ social and emotional lives [35,36]. Patients’ identities are called into question as they repeatedly face uncertainty and ambiguity regarding their condition and its prognosis. As they experience chronic sorrow about their condition, and as they are repeatedly stigmatized as a result of their disability, the patient grieves the losses in their identity associated with and resulting from these factors [36,84,85,86].

Patients may experience grief from the loss of their previous life, including their previous roles and relationships. Indeed, patients with ME/CFS (as well as those with fibromyalgia) reported more loss due to their illness than did patients with other chronically fatiguing diseases: more loss of social support by family and friends, more loss of recreational activities, and more loss of material possessions [92]. The subsequent grief from this variety of losses is often prolonged due to the chronicity of their condition. However, it is important to note that because grief is usually associated with death-related losses, patients are more apt to have their bereavement disenfranchised by societal reactions [93]. As a result, resolution is a difficult goal for patients experiencing grief. The cyclical nature of ME/CFS means that there is no way to simply “move past” the loss, and because it is repeatedly experienced, patients’ normal grief responses are often pathologized and disenfranchised [2,93].

#### 3.3.3. Social-Interactive Domain

In Phase 2, ME/CFS patients encounter increased conflict as friends, family members, coworkers, and some care providers can lose patience. By and large, society’s model for illness is that of an acute condition. People are usually tolerant of ME/CFS patients when they first become sick, but this is with the expectation that they will eventually be cured and return to their normal functioning. The persistence of symptoms can predictably frustrate the patients’ support networks. 

As a result, patients may face several limitations in the roles they once occupied in their pre-crisis lives, as parents, employees, community leaders, and so forth. Role confusion can occur in patients as their position within their family changes, such as with the case of a mother, for example, who is too fatigued to care for her son, a husband who is unable to find joy in his relationship due to the intrusiveness of his condition, or a parent whose financial and functional losses have resulted in an inability to provide a sufficient income for their family [2,92,94]. 

Coupled with a chronic illness experience is the loss of various social aspects of the patient’s old life that can no longer be recovered. For example, physical symptomatology, such as post-exertional malaise and pain-related sequelae, may significantly impact certain patients so that they experience a loss in their ability to maintain romantic and sexual relationships. Meanwhile, sleep-related disturbances and fatigue may significantly intrude in certain patients’ daily energy expenditure, resulting in a loss in their ability to maintain platonic relationships [6,38]. Inevitably, patients’ inability to fill roles they did prior to their illness experience can initiate or exacerbate trauma symptoms and cumulatively increase the burden put on them by their illness [92,95].

Oftentimes, the friends, family, and romantic partners—a group that makes up the support system of an individual—may share the patient’s grief as well. Caregivers experience their own set of fears, anxieties, and even grief over the various aspects of the patient that have changed due to ME/CFS. Several lines of research support the vulnerability, frustration, guilt, uncertainty, loss of identity/role confusion, functional and financial losses, and lack of professional support in caregivers of chronically ill patients [83,96,97]. There is an unfortunate feedback loop here as patients perceive they may be a burden to their support system and this may worsen the patients’ psychological symptoms.

The response to ME/CFS can dramatically alter the lifestyles, finances and work habits of spouses and parents. The life they had or had been working toward may not be possible now. Vacations, further education, marriage, or children may be indefinitely postponed. Families frequently divorce under the strain. Part of their frustration stems from their own experience of vicarious secondary wounding. Research suggests that populations that frequently and repeatedly engage with the traumatized individual, a group that can vary from family and romantic partners to the medical professionals and clinicians that treat them, can experience vicarious traumatization [61,65]. Oftentimes, even when the patient is able to receive help with their trauma, those vicariously traumatized are at a loss [83].

Friends and family can also suffer “guilt by association,” that is, the family is stigmatized for the patient’s illness. Even clinicians can find themselves stigmatized when they treat the ME/CFS population. As a result, it is not at all uncommon for significant others to depart sometime during Phase 2, which can create feelings of betrayal and trauma in the patient [87]. As ME/CFS patients face increasing difficulties with their support network, they begin to actively seek out a new network of friends and more information about their illness [98,99,100,101]. It is often among these others of “like kind” that patients begin to establish a nucleus for a new community of supporters who will accept them as they are, disabled with ME/CFS [2,3].

Patients who are severely or very severely affected are usually not employed when they are in Phase 2 and this can add to the financial stress that may have begun in Phase 1. If they are, they usually find their functioning at work stabilizes at the same time as their physical symptoms. Typically, these are individuals who can work from home on a very part time basis with no set schedule. In many cases, if possible, they ask for a leave of absence or take sick leave because they hope that a cure will allow them to return to full-time employment. Others ask for part-time work, quit their jobs, are asked to resign, or are fired. This adds a serious financial concern to all the other problems of ME/CFS. Adding to the financial strain is the cost of the increased medical care itself; research shows medical costs to be 50% higher for patients with ME/CFS as compared to patients with lupus or multiple sclerosis and three to four times higher than the average insured person [102].

### 3.4. Elizabeth’s Story: Phase 2

#### 3.4.1. Physical-Behavioral Domain

During Phase 2, Elizabeth attempts to carve order out of chaos. Her physical situation is very limited but seems to have stabilized. Only a few new symptoms have suddenly surprised her. None of her present symptoms seem to be getting progressively worse, at least for now. Her symptoms do not disappear, but they usually do not exceed patterns that she is beginning to decipher. If she has an especially bad night sleeping, she knows that she will probably have an especially bad day. She will have more pain and more cognitive confusion. If she does two hours of steady activity, she knows that her body aches are likely to increase and her glands will probably swell. Life is very difficult, but Elizabeth has identified a set of parameters around which she can function. Her health care professionals discuss a few of these parameters with her, but for the most part Elizabeth discovers them on her own. To some extent, her newfound knowledge also orients the people around her.

While in Phase 2, Elizabeth suffers two significant physical relapses beyond her usual baseline of functioning. Each time, she suddenly becomes far more exhausted than usual. She cannot even lift herself out of bed without physical assistance but feels as though she is being pulled down through her bed toward the center of the earth. Her head aches, her glands are very swollen, she has nausea, and she becomes intolerant of light and sound. She cannot organize her thinking at all. However, both times she relapses, she eventually returns to a plateau of stabilized symptoms that she recognizes and can negotiate.

#### 3.4.2. Psychological Domain

Over time, Elizabeth’s physician becomes increasingly convinced that the tentative diagnosis of ME/CFS does indeed fit her symptoms, and thus, her tentative diagnosis is confirmed. Initially, she feels enormous relief. Finally, she has an explanation for why she is so exhausted, why she cannot sleep, why she has muscle pain and headaches, and why she sometimes becomes cognitively confused. Her uncertainties also lessen as she begins to recognize her symptom pattern. Furthermore, diagnosis gives her a framework to learn about her condition so that she can exert a semblance of control over her life again. When she can read, she reads everything she can about ME/CFS and seeks out others with ME/CFS so that she can discuss her situation in a supportive setting.

However, Elizabeth quickly learns that the diagnosis does not explain how her illness started or what is going to happen in the future, so painful uncertainty and ambiguity return. No one seems to know what to do to cure her. No one can make her symptoms stop, and no one seems able to tell her how she is supposed to live her life under these conditions.

Elizabeth grew up believing that if she worked hard and told the truth, everything would eventually come out all right in her life. However, here she is, working hard to get better, telling the truth when she talks to family members and friends and clinicians, and yet a significant percentage of the time she finds not acceptance, but rejection, from many people. In fact, she is sometimes being blamed. So Elizabeth has become extremely cautious. She carefully censors what she says and to whom, and whenever she possibly can and for limited periods of time, Elizabeth carries on as though she is well and nothing is wrong with her. Elizabeth decides to avoid such secondary wounding by withdrawing from any social contacts that may evoke negative judgments. Instead, she tries to get in touch with other ME/CFS patients and ME/CFS advocates because they are likely to be helpful and will understand her situation. She continues to read about her condition and seeks sources of emotional sustenance to try and make up for the multiple losses she has suffered.

Because her medical outcome is uncertain, and Elizabeth still believes that a cure must be a possibility, she suspects that her health care professionals are not adequate to deal with her problem. She collects the names of other doctors from friends and new ME/CFS acquaintances and attempts to find a professional who will offer her better treatment and, she hopes, a cure. 

Unfortunately, Elizabeth finds limited guidance and meets with confusing responses and even outright hostility as she consults other doctors, so she attempts alternative treatments. A practitioner of shiatsu massage listens to her with enormous empathetic patience, but she cannot continue to afford the sessions, and it is unclear if they are helpful. Elizabeth’s cousin urges her to try acupuncture. A former coworker swears that a complicated vitamin and supplement regime returned her bedridden niece to full functioning and would do the same for Elizabeth.

Elizabeth has lost a sense of her boundaries. To others, she seems to have given up and they are confused, not grasping how exhausting it is for her to remain in ongoing contact. Elizabeth’s family and employer are encouraging, even urging, her to return to her former roles and schedules. However, Elizabeth‘s efforts to return to these roles can have dangerous repercussions. She has trouble getting up in the morning and doing her basic activities of daily living. She showers weekly and washes her hair bi-weekly. Her husband has to make the children’s school lunches. She can no longer serve on committees at her children’s school because she can just barely keep up with basic self-care. In fact, she rarely leaves the house and only when her husband or a friend is driving. Her last attempt behind the wheel left her terrified and exhausted after a near miss at a stop sign she had not realized she had run through. Nothing about her body or her emotions or her mind acts the way it did in the past, and yet Elizabeth keeps trying to behave as though she were the person she used to be. Despite her efforts, Elizabeth fails daily at what she attempts, and daily she feels guilty and ashamed. Increasingly, she feels worthless.

#### 3.4.3. Social-Interactive Domain

Elizabeth experiences growing conflicts with family, friends, and some of her medical care providers as they lose patience with her failure to become symptom-free or to adjust to her illness in a way that allows her to return to her former functioning. Although she has a diagnosis, such treatment as she receives does not produce rapid, let alone any significant, improvement. At one point when she was barely sleeping, Elizabeth’s doctor put her on a course of medication. She somewhat improved, but she still has consistent difficulties.

Jim has told Elizabeth that she is no longer the person he married and this is not the life he signed up for. She has got to change if their marriage is to continue. Sex has stopped and she barely has the energy to regularly watch a movie with him. Elizabeth can tell that her coworkers are annoyed and believe that she could function a lot better if she just pulled herself together and put her mind on the job. One of them knows a ME/CFS patient of moderate functioning and tells others at work that she cannot understand why Elizabeth does not manage as well as her friend does. Elizabeth is not imperceptive. She knows that people think she is not trying hard enough. To make matters worse, a close friend with deep religious convictions has urged Elizabeth to pray, saying that if Elizabeth has a sincere desire to get better and asks for God’s help, God will cure her. Elizabeth does not share her friend’s convictions, but deep inside she fears that maybe she is sick because she is somehow unsatisfactory in God’s eyes.

As Elizabeth goes through relapses, all the people in her life experience them as well. They become as exhausted by the process as Elizabeth does, and they are traumatized just as she is. Jim has lost the wife he married and the life he had, and his new life is not at all what he wants. Their son, Michael, has always liked school and has done well, but now his grades are beginning to suffer and it is difficult to participate in sports since his mom cannot drive anymore. Eva is behaving badly at home, and she has been acting out, and repeatedly disciplined at school. Both kids complain that their mom never comes to their soccer or baseball games and are embarrassed to rely on friends’ parents to drive them. Elizabeth does not know how much of this is just a part of adolescence, or whether Eva and Michael are reacting to her health problems and her arguing with Jim over money, the division of labor at home, and her condition. Elizabeth’s husband and children are not mean spirited. They are sad and scared to see this person who is very important to them suffer pain, confusion, and unhappiness. Outside the house, they suffer a kind of guilt by association. Eva’s friends sometimes treat her as though she is as weird as her mother, and Eva overheard one of them say that Eva’s mom was an alcoholic who was always hungover. Jim’s boss is clearly concerned about whether Jim will be able to fulfill his job obligations, given the demands of Elizabeth’s illness. Some secretly wonder whether ME/CFS might be contagious and just for safety’s sake, many keep their distance.

Even Elizabeth’s doctor is affected. Some clinicians who treat ME/CFS patients report that colleagues are skeptical when making referrals. Specialists in ME/CFS sometimes even meet skepticism in social settings. A neurologist to whom Elizabeth’s doctor was going to refer her said that he did not think ME/CFS was a valid diagnosis and that he believed Elizabeth was suffering from a psychological condition.

As normalization failure is so common in Phase 2 [2,3], like the chronically ill person, those around them may turn to alcohol or drugs. People in the social network may avoid or even abuse the chronically ill individual. Any of these factors, or a totally new factor, can produce another crisis in the chronically ill, returning them to Phase 1. Jim’s mother dies and the entire family must deal with that loss. Later, Elizabeth has a high fever during a bout with the flu that triggers a severe relapse.

Elizabeth is a fortunate person with chronic illness in that she has some warm and loyal friends. Her supervisor persuades management to let Elizabeth try working remotely with just a few hours a week doing billing as she is able. Additionally, a social worker newly affiliated with Elizabeth’s doctor’s office becomes involved in helping Elizabeth cope with ME/CFS.

### 3.5. Phase 3: Resolution

Without informed clinical guidance, many chronically ill people become caught in a repeating cycle of Phase 1 and Phase 2 [2,3]. Each new crisis produces new wounding and secondary wounding [55,72,74]. With luck, following each crisis the patient manages to arrive at a plateau of recognizable symptoms, until the next crisis sends the whole system into chaos again. Some people, particularly those on the margins of society who have almost no sources of support, never escape Phase 1, but are buffeted from crisis to crisis.

The personal, familial, and societal pressures heaped upon the patient to return to the pre-crisis life are enormous and can help maintain a cycle between Phase 1 and Phase 2. Most patients present to clinicians in Phase 1 or Phase 2. Repeatedly, they are urged by family and friends to try the next “cure”, and in their growing desperation frequently do so. The clinicians also feel the omnipresent pressures to repeatedly treat the chronic patient. This approach will ultimately fail, frustrating all parties involved. Because they are all still struggling in the chaos of the crisis phase, or they are in the stabilization phase and have achieved some equanimity, however brief, they are not ready to grasp that it is highly improbable that they will return to the pre-crisis life and that they have to transition to a new life—a new way of being in the world.

This is the universal clinical treatment problem between Phase 2 and Phases 3 and 4: how to facilitate this painful transition for what is actually possible with a severe disabling chronic condition; otherwise patients never escape the futility of a Phases 1 and 2 cycle. Without an understanding of the nature, breadth, and depth of ME/CFS suffering and without a grasp of the longitudinal, cyclical experience of the phases, health care professionals will find it difficult to compassionately assess and treat ME/CFS patients. Important opportunities for a better, albeit a different life, can be missed.

#### 3.5.1. Physical-Behavioral Domain

Most Phase 3 ME/CFS patients who are severely or very severely affected maintain a continued plateau, but relapses occur. Sometimes old symptoms worsen or new symptoms appear. Some ME/CFS patients experience modest periods of improvement and some have learned to balance activities to keep from relapsing [13,94]. If a relapse takes place, it is sometimes in response to the typical cycling of ME/CFS symptoms, but it may also be triggered by persistent attempts to engage in pre-crisis tasks, roles, and pursuits. True entry into Phase 3 comes, however, when ME/CFS patients recognize that they cannot perform as they used to in the past. Table 3 lists the stages and characteristics of Phase 3.

#### 3.5.2. Psychological Domain

ME/CFS patients in Phase 3 suffer a secondary emotional crisis or grief reaction when they acknowledge the chronic nature of their condition. They finally realize that their lives have changed forever, and they begin the process of mourning their pre-crisis self. They typically feel demoralized and devalued, for they see that they can no longer carry out their previous roles in life—as parent, worker, lover, friend—in the way they had always thought they would. They may question what good they are, who they are, and why they should continue to exist at all. This appropriate, necessary grief reaction—their “dark night of the soul”—is a tenuous time. Individuals can be lost in their own understandable withdrawal, fall victim to predatory providers, or succumb to despair and thoughts of suicide [2]. Research suggests a relationship between suicidal ideation and illness identity [103,104], and as patients grieve losses that have resulted from their illness, the negative identity associated with the illness can be worsened. Yanos and colleagues [104] propose that patients’ conceptualization of their illness identity can affect their hope and self-esteem, which further increases the likelihood of suicide. Given the susceptibility of ME/CFS patients to experience threats to their identity and role confusion, afflicted patients who feel stigmatized may turn to suicide due to a reduction in their self-esteem. Indeed, evidence supports this conclusion, as suicide has been identified as a cause of death for a significant number of ME/CFS patients [105,106]. Additionally, patients who commit suicide appear to lack sufficient depressive symptoms to qualify for a depression diagnosis, as their depressive symptoms may better be associated with grief [107]. Evidence suggests that losses associated with ME/CFS and perceived stigma may be likely risk factors for suicidal ideation [42]. Individuals may be traumatized at various points during this often cyclical process.

If, however, the patient with ME/CFS can work through this existential angst and establish meaning in their lives, they can then take marked steps toward constructing an authentic new self and a new life [84]. Indeed, individuals with a chronic illness or disability report higher post-traumatic growth than do those without a chronic illness or disability [108]. Many patients report new insight due to their illness experience [90] and some reported having a more confident and assertive personality as a result of having ME/CFS [16]. However, post-traumatic growth was found to be lower among women with ME/CFS than among women with rheumatoid arthritis, osteoarthritis, and multiple sclerosis, perhaps because of the greater levels of stigma women with ME/CFS felt due to having an unexplained illness [109]. 

Meaning is established over the phase process through three transformational steps: (1) the allowance of suffering as opposed to its rejection and the subsequent rejection of the suffering self; (2) the development of a compassionate response to the suffering of the rejected, sick, stigmatized self; and (3) the development of respect for their suffering and their ability to live with it and despite it. Creative activity is a successful path leading to the creation of meaning [108]. So, too, is a sustaining faith on the part of the care provider that the ME/CFS patient can construct an authentic new self.

As ME/CFS patients move through Phase 3, they develop a strongly internalized locus of control and increased tolerance for the uncertainty, ambiguity and chronicity of ME/CFS. They openly express compassion for themselves, and they begin to reconstruct an illness narrative that eliminates the harmful social messages they have endured until now. Their work to achieve meaning involves them in philosophical or spiritual development that offers an ongoing framework for adapting to new experiences, whether good or bad.

#### 3.5.3. Social-Interactive Domain

Despite improvement in the psychological domain in Phase 3, ME/CFS patients may undergo even greater losses than they did in Phase 2 as significant others depart, clinicians give up, and friends disappear. They may continue to experience abandonment, isolation, and stigmatization. Associated with these personal losses are the subsequent losses in romantic partners, friendships, and even close family that deepens the patient’s negative evaluation of their condition. A combination of the uncertainty and ambiguity they encounter [88,89,110] and the losses they experience chip away at the patient until they slip into another form of chronicity in their sorrow [111]. These losses are cumulative and compounding in nature; each new loss is another weight that produces and maintains grief. 

At the same time, as they are able, they continue to pursue new friendships and the support of others with ME/CFS. Some Phase 3 patients can still feel engulfed in stigma, particularly when accompanied with the fear that their condition could be “exposed” to the public, further exacerbating their depressive symptoms and engagement with treatment [31,41,43,112]. Others may not, but neither group wants to remain silent. They are more willing to speak up against stigmatization and confront it instead of avoiding persons who behave inappropriately toward them. As they work through their existential issues and begin constructing a new authentic self, they begin experimenting with new social roles and sometimes, if able, with new part-time jobs or vocations. Some may engage in social or political activism related to ME/CFS [2,3].

### 3.6. Elizabeth’s Story: Phase 3

#### 3.6.1. Physical-Behavioral Domain

In Phase 3, Elizabeth experiences periods of stabilized symptoms, sometimes even minor improvement, but she still has relapses. Most of these are simply in the nature of ME/CFS. As Elizabeth comes to comprehend the chronicity, ambiguity, and uncertainty inherent in her condition, she lets go of her search for an elusive cure and works instead to integrate her illness into a new life.

#### 3.6.2. Psychological Domain

Twice in Phase 2, Elizabeth suffered severe relapses brought about, in part, by her repeated attempts to do some of the things she did before her illness. Throughout that time, she wanted to be her former self, and everyone around her wanted her to be that person, too. However, repeated relapses have taught her that she cannot sustain the roles that she had always thought she would fulfill as spouse, parent, worker, or friend, or at least not in the way she used to imagine. Elizabeth comes to understand that her life has changed entirely and forever.

With the help and encouragement of her new ME/CFS friends and the social worker, she explores and expresses the grief she feels for the loss of her old self and she mourns the end of that life.

At this point, all the major existential questions come into play. Elizabeth wonders, “Who am I?” “What good am I?” “Why did this happen to me?” “Why should I live?” “Is there any value to my life?” During this painful period, she struggles to locate a meaning for her existence and her suffering. Elizabeth is very vulnerable. She could be lost because of her considerable social withdrawal. She could fall victim to cynical and predatory providers. She could give way to despair and attempt to kill herself.

However, again, Elizabeth is fortunate. Her new friends and the social worker help Elizabeth navigate the difficult course between necessary grieving for her past self and floundering in grief and reactive depression. She learns not to reject but to allow her new suffering self and to have compassion for herself and her suffering as well. This is not an easy task because Elizabeth is constantly receiving messages from people she knows telling her that if she stays the way she is, if she remains ill, then they no longer want her among them.

To move forward from grief and mourning, Elizabeth tries to discover meaning for what has happened to her and locate a way to live in the future. She begins to engage in philosophical or spiritual thinking in order to come to a new place. Elizabeth starts by learning to respect the person she is now—not the person she might become, but who she is right now.

As is typically the case, Elizabeth does this through a creative act that becomes an act of meaning development. She decides to write a journal describing her experiences. Other people she knows have done things as various as taking up painting, becoming ME/CFS advocates, even conducting online support groups. In composing her journal, Elizabeth re-creates herself—she integrates herself and begins to discover meaning in her experience.

Elizabeth draws heavily on her social worker’s clinical skills, personal support, and encouragement and on a variety of wisdom traditions that seem to speak to her. Elizabeth is not religious in the traditional sense. However, since she has consciously begun thinking about the basic issues of life and meaning, she has discovered aspects of Buddhism and Celtic philosophy that resonate strongly with her personal vision of what is significant in life.

#### 3.6.3. Social-Interactive Domain

The strides Elizabeth makes in her psychological evolution during Phase 3 do not occur in a benignly static social environment. She endures a considerable blow when Jim tells her that he wants a divorce and he has a new relationship. At first, Elizabeth worries terribly about how she will manage the loneliness and the bills. She still has a very limited job at the medical practice and she has always been on Jim’s health plan. As part of the divorce settlement, he agrees to keep her and the children covered. He is also good about having the children visit regularly, which gives Elizabeth needed quiet time and reduces her anxiety that the divorce will cause harmful distance between the children and their father. Elizabeth knows that in this divorce experience, she is much more fortunate than one of her friends with ME/CFS who lost custody of her children and lost her home when she subsequently lost her job.

Encouraged by a ME/CFS friend, Elizabeth explicitly asks Eva and Michael for help at home. To her surprise, Eva responds enthusiastically, especially to cooking. Michael is good about drying dishes and starting a load of dirty clothes in the washer if reminded, but he has recently expressed wanting to live with his dad. Elizabeth, who could not have endured letting him go two years ago, now feels confident about his basic attachment to her and is planning to let him spend his next school year with his father.

At the medical practice, Elizabeth usually feels able to deal with her present limited job requirements. Her coworkers, to the extent they are aware, have gotten used to her condition and have more or less forgotten it. Her confidence was further bolstered when the social worker offered to conduct a workplace consultation for disability accommodation on her behalf. Elizabeth decided that it was not necessary at the present time, but she felt she had someone on her side if she should need it.

Elizabeth knows, however, that her job security depends almost entirely on her supervisor, and she has begun investigating other part-time work she might do from home. The social worker has also reminded Elizabeth that she is eligible for disability, and she has been inquiring among her ME/CFS friends about this as well.

In any social arena now, Elizabeth is less likely to keep silent about her illness. When people react badly or seek to label or stigmatize her, she may confront them about their bias. She has been surprised at how empowered such behavior makes her feel, and she is even thinking about becoming formally involved in advocacy work. The end of her marriage and the inevitable loss of some old friends and acquaintances have forced Elizabeth to consider new roles and to seek new friends. Although this experience has been intensely painful, Elizabeth continues to survive it. She is surprised to find how the process has turned out and how she is adjusting to her new self.

As Elizabeth freely acknowledges, it would have been very hard for her to navigate this passage without the informed help of her care providers—her primary care physician, the specialists, her friends, and the social worker. For example, the social worker suggested a number of books that she thought might help Elizabeth think about the philosophical questions involved and put her in touch with a social work expert in issues of major loss and trauma who helped Elizabeth discover what problems had bedrock significance for her.

### 3.7. Phase 4: Integration

#### 3.7.1. Physical-Behavioral Domain

ME/CFS patients in Phase 4 may experience physiological plateau, improvement, or relapse. By this time, ME/CFS patients recognize the cyclic nature of their illness and no longer regard relapse as failure. Relapse is simply the beginning of another cycle that they must integrate. This understanding manifests the true nature of acceptance for ME/CFS patients, which is the integration of their ME/CFS into an ongoing, full life. When patients experience new symptoms or familiar ones worsen, they contact their health care providers immediately. Otherwise, they maintain a sensible monitoring of their condition and see clinicians on whatever schedules their clinicians have advised. Table 4 lists the characteristics of Phase 4.

#### 3.7.2. Psychological Domain

Phase 4 ME/CFS patients have achieved a true integration of the pre-crisis self with the newly claimed respected self who has suffered and endured, similar to the “recovery in” framework discussed by Devendorf and colleagues [42]. They maintain this achievement through a daily commitment to allowing their suffering, meeting it with compassion, and treating it with respect. Life for them will necessarily include small daily acts of bravery in the presence of stigmatization, rejection, or the pains of ME/CFS itself. Patients do this in an exercise of free will, not because they are forced to. They formulate and then live up to a new “personal best.” They continue to work on meaning development in conjunction with their continuing pursuit of philosophical or spiritual development. 

#### 3.7.3. Social-Interactive Domain

In Phase 4, ME/CFS patients, as they are able, continue to be involved in creative and social action. They expand their circle of supporters, but their increased self-assurance and self-confidence also permits them to attempt reintegrating old supporters who fell away in the past. They are often willing and able to help these people learn, if these old friends wish to be reintegrated. Although most severely and very severely affected ME/CFS patients have difficulty maintaining any work schedule, some of them, especially in Phase 4, find vocational activities that allow them to participate to the limit of their abilities in activities that they value.

### 3.8. Elizabeth’s Story: Phase 4

#### 3.8.1. Physical-Behavioral Domain

For the most part, Elizabeth experiences continuous plateau, and she enjoys occasional, limited improvement. However, she has also had three relapses—one severe and two lesser ones. Elizabeth now realizes that relapses happen, and she no longer regards them as some failure on her part. However, short of death, which will happen to everyone eventually, she intends to try to reintegrate herself after each relapse experience. Elizabeth comprehends that integration is the “philosophy of life” she should strive to maintain.

#### 3.8.2. Psychological Domain

Elizabeth maintains her new self by consciously recognizing who she is now and by standing with herself. This does not mean that life has become easy. Frequently, Elizabeth cannot climb stairs at all. Sometimes she is so debilitated she must use a wheelchair at home, which she hates. She can still become cognitively confused, especially if she overextends. She still experiences some difficult moments of stigmatization and rejection, and even her own pain. However, she often speaks out against bias and has learned to endure the symptoms of her illness. She has created a new ideal self and takes pleasure in seeing how well she can live up to it.

Elizabeth finds that a constant, active, conscious consideration of meaning and purpose enriches her life and places her experiences, both positive and negative, in a context larger than herself. She still finds great solace in Buddhist conceptions of suffering, but she has also discovered a new trove of wisdom in the material discussed in an online Great Books virtual discussion group that she has joined.

#### 3.8.3. Social-Interactive Domain

Elizabeth continues to nurture the new friendships that she began establishing in Phase 3. She also, through social media, sought out her younger sister, from whom she had been estranged as an adult, and the two have found they enjoy the openness and honesty of their new relationship as much as they like reminiscing about their childhood. Elizabeth’s frankness about her condition and her refusal to accept derogatory estimations make it perfectly clear to people who she is now, and some admire her for it and see the truth of her self-assessment.

Elizabeth has changed her job situation. With her social worker’s assistance, Elizabeth is receiving disability and is being accommodated at her very part time, remote job. Although Jim has remarried—an event that threw Elizabeth into an emotional crisis—they have remained civil, though distant, friends. Relations between the two are better than they have been for many years.

Elizabeth even dares to contemplate entering a meaningful sexual relationship again. One of her ME/CFS friends began online dating recently, which gives her hope, and she has met a man she likes very much in an online writing class. Because of his encouragement, she sent part of her journal to a ME/CFS patient advocacy website.

Elizabeth knows that crises and disasters happen all the time in life. She worries a lot about her children. One of her ME/CFS friends took a terrible turn for the worse and has been completely bedridden for 3 months. This scares Elizabeth terribly, for she knows the same could happen to her. However, Elizabeth is learning to separate those things she can control from those she cannot. Although it is a continuous effort, she endeavors to exert herself for the things she can affect and to endure with grace those she cannot.

## 4. Intersectionality

Examining Elizabeth’s life with severe ME/CFS as a white woman does not capture the experiences of men, people of color, people of lower socioeconomic status (SES), or people who are in the lesbian, gay, bisexual, transgender, and queer community. For example, men with ME/CFS and other chronic illnesses may have additional issues to work through given the stereotype that men are supposed to be strong [86,113]. Additionally, it is important that appropriate attention is paid toward communities that are often overlooked in discussions of ME/CFS. Increasingly, evidence suggests that ME/CFS has a higher prevalence in communities of color [114,115]. Health disparities between white and minority American women are well documented in the literature [116], with poorer self-perceived health statuses in African American and Latina women, limitations in activity levels due to health, and the inability to work as a result of those limitations [117]. In their qualitative study, Bayliss and colleagues [118] highlight some profound aspects of what it is like being a minority navigating health care. Several participants shared concerns over a lack of communication, for example, that may be more relevant for communities of color. Indeed, evidence suggests that the patient’s language preference plays a role in their access to health care [119,120]. Furthermore, participants in Baylis and colleague’s [118] study also discussed discrimination and stereotyping when seeking treatment for ME/CFS. As patients of color face multiple hurdles in their attempts to seek treatment, their lack of treatment compounds with the coexisting hurdles they face as minorities.

Along with race, SES has been shown to be a risk factor for more severe presentations of ME/CFS [121]. This can be exacerbated when low SES patients experience difficulties in employment due to their condition. For example, patients who are homebound with ME/CFS are more likely to be unemployed as a result of their health status [34] and thus can suffer from decreases in SES as a result of their illness. 

The multiple, compounding adversities that plague people of color can have a profound impact on their symptomatology and well-being. Turner and colleagues [122] posit the concept of cumulative stress burden in an attempt to operationalize the burden that affects an individual’s health. It is therefore likely that patients who experience greater stress, such as patients of color and patients of low SES, may be at higher risk of stress-related health problems. Research suggests that there is an association between cumulative stress and health in various communities. Discrimination in African American and Latin American communities acts as a contributor to poor mental health. Both of these communities have been found to have high levels of depression along with moderate levels of anxiety and post-traumatic stress disorder [123]. Concurrent life events such as natural disasters also impact health and exacerbate health care disparities when there is a lack of protection of groups with compromised health [124], and as communities of color lack health care protections at a disproportionate scale [125], they too are at risk for cumulative stress-related concerns. Significant stressful disruptions can cause patients to re-enter the crisis phase before resuming the chronicity of their condition; such an occurrence may heighten the probability of traumatization and disenfranchise their grief. Thus, the debilitating nature of ME/CFS can have a particularly intrusive effect within populations that lack the protective factors that higher SES and racial majorities can have. Being severely and very severely affected with ME/CFS can be yet another barrier for those people of color and those with lower SES.

## 5. Utilizing the Four Phase Model: Managing Suffering, Chronicity, Uncertainty, Ambiguity, and Severity

The Four-Phase Model attempts to shift the focus of the standard care paradigm for chronically ill individuals. Most obviously, it moves from a unidirectional, acute model of illness to a conception that encompasses the cyclic nature of chronic conditions. The Four-Phase Model maps a process that most individuals do not enter into willingly. It recognizes that for patients with any chronic condition, the situation in their lives is *imposed*. The Four-Phase Model is also characterized by a broad inclusiveness. It addresses from a systems perspective the total environment of a patient’s life, including the physical, psychological, and social domains. Its approach is distinctive in that it considers the physical and psychological domains as having equal importance with each other and with family and work-related issues. In actual experience, once patients have acquired methods for maintaining the best level of physical well-being they can, most chronic illness patients cite social and economic issues as causing them the greatest difficulties.

The model incorporates uncertainty and ambiguity as essential, irreducible aspects of chronic illness. It posits an integrated, meaningful life as the end sought in patient treatment, rather than a cure, which, by the definition of chronic illness, does not exist. Most people with chronic conditions will not experience cure; hence, they are better served by a paradigm that organizes itself around the uncertainty, chronicity, ambiguity and severity that reflects their actual situation. To this end, it is vitally important to recognize that the suffering engendered by the severely and very severely affected is dramatically increased, frequently to the extreme.

The model identifies four distinct phases in the illness experience of patients, though these phases do not follow an irreversible progression forward. Rather, the model acknowledges the cyclic pattern of the chronic illness experience and incorporates the concept that severe relapses, other illnesses, or non-illness-related crises may return patients temporarily to earlier phases. The Four-Phase Model asserts that patients who make a thoughtful, reflective progression through all four phases will have the knowledge, understanding, and techniques for moving through the phases more quickly a subsequent time, although the degree of severity that the patient experiences impacts the process. They will also have deeply assimilated the concept that it is possible for them to achieve a better life even within the limitations of their situation, and even if that situation is severe or worsens. 

The Four-Phase Model presents what may be expected over time and indicates appropriate times and ways to intervene to improve the patient’s quality of life by matching intervention to phase. Conversely, attempting interventions at the wrong time may prove less effective and may undermine the possibility of the same interventions being effective at a later phase when the patient would ordinarily be receptive to such interventions. The Four-Phase Model also suggests comprehensive interventions that take into account interactions among the multiple domains in which patients experience ME/CFS.

### 5.1. Use of Standard Interventions and Current Therapies

Adopting this model does not require clinicians to learn an entirely new battery of interventions, but to supplement those already in use with new techniques. The Four-Phase Model does not present an exclusive form of assessment and treatment. It complements standard assessment tools and current therapeutic methods with additional investigatory tools and interventions. It also covers the entire range of the patient’s life experience, not only the patient’s medicalized body or psyche. The model also frequently incorporates existing interventions that clinicians currently use. The techniques of stress management and of post-traumatic stress disorder (PTSD) reduction and management, for example, are particularly useful with Phase 1 patients who need to reduce the chaos of their experience before they can move forward. Many aspects of occupational therapy work well, particularly with Phase 2 patients as they try to gain insight into their activities and change habits that undermine the level of health that they can maintain. A host of grief therapies, meaning development, and art therapy techniques are especially useful during Phase 3, and the well-known benefits of journaling contributes to the writing and rewriting of the illness narrative, which is a distinctive feature of therapy throughout the four phases.

By serving as a narrative or cognitive map, the phase description helps to lessen the intense fear and anxiety frequently experienced by the severe and very severely affected ME/CFS patients and their families. It will also help them to know they are not alone, their experience is shared by others, and they are understood. They now have a method of validating their experiences and making them known to others. The narrative helps them develop a sense of what is happening to them and provides a degree of order and coherency about their illness experience. In addition, the mapping aspect of the phase process helps promote understanding and adjustment to the cognitive impairments in concentration, memory, and decision making that often affect those with ME/CFS.

### 5.2. How Health Care Professional Can Help

While pilot assessment and treatment programs, in all domains, are underway worldwide, patients, their families and friends live and suffer with ME/CFS. They struggle to have whole lives and clinicians and caregivers struggle to help them manage. How can health care professionals help?

During the brief time available in a patient visit, here are some important things health care professionals can do, in addition to the medical protocols:

- Demonstrate to patients an appreciation and understanding of the ME/CFS experience;
- Convey to patients the compassion that comes from an appreciation of what the severely and very severely affected patient is experiencing;
- Communicate to patients that you *believe* what they are saying about their experiences and symptoms;
- As time and opportunity permits, be open to learning more about this poorly understood syndrome;
- Have available a short list of therapists and specialists, including those who do trauma work, grief work, family therapy, couples therapy, sleep hygiene and occupational therapy, for referral, or to be part of the treatment team;
- Become familiar with the suicide hotline;
- Have available a list of patient support groups for information, education and support;
- Have a list of available support groups and assistance for caregivers;
- Remember that it is very difficult to care for those who do not recover in any conventional sense and clinician resources for support are always a good consideration as well.

Severely and very severely affected patients suffer profoundly. In order for health care professionals to adequately treat their patients, they need to understand all that composes and creates their suffering: struggling with uncertainty, ambiguity, chronicity, stigmatization, trauma, and rejection. These elements create losses for the patient and they subsequently grieve these many and varied losses, including lost friends, family, career, and life as they knew it (or imagined it). Not only do the patients grieve their losses and traumas, but so do the loved ones around them—spouses, parents, and children. Thus, in order to assess and treat, the suffering must first be described, understood, witnessed, and, most importantly, abided.

## Figures and Tables

**Table 1:** Phase 1: Crisis.

**Physical-Behavioral** Coping period Onset period Acute emergency period **Psychological** Loss of identity and/or loss of psychological control Intrusive shame, poor self-esteem, despair Shock, disorientation, dissociation Fear of others, isolation, emotional lability **Social-Interactive** Others experience shock, disbelief, and/or revulsion Vicarious traumatization Family and organizational coping Others fall within a continuum from suspicion to support

**Table 2:** Phase 2: Stabilization

**Physical-Behavioral** Plateau Stabilization **Psychological** Increased caution and fear of secondary traumatization and wounding Social withdrawal from prior social circles; social searching for others with ME/CFS Medical/clinical service confusion; searching for appropriate and compassionate care Boundary and role confusion **Social-Interactive** Interactive conflict or cooperation from others Vicarious secondary wounding Vicarious traumatic manifestation Social normalization failure

**Table 3:** Phase 3: Resolution

**Physical-Behavioral** Patients differ in their experiences, ranging from (a) continued plateau/stabilization/improvement, (b) emergency period or diminished functioning, or (c) relapse **Psychological** Grief reaction and/or compassionate response to self Identifies with pre-crisis self Role and identity experimentation Returning internal locus of control Awareness of societal effects Spiritual development **Social-Interactive** Breaking silence regarding disbelief of ME/CFS and stigmatization Confrontation regarding care and social roles Social and vocational role experimentation Potential integration or separation or loss of supporters

**Table 4:** Phase 4: Integration

**Physical-Behavioral** Possible continued plateau or improvement or relapse Recovery period **Psychological** Role and identity integration New personal best Continued spiritual and emotional development **Social-Interactive** New and reintegrated supporters Alternative vocation and activities

## Data Availability

Not applicable.
